# A novel combination of an IgE mediated adult onset food allergy and a suspected mast cell activation syndrome presenting as anaphylaxis

**DOI:** 10.1186/s13223-016-0151-z

**Published:** 2016-09-17

**Authors:** Colin Barber, Chrystyna Kalicinsky

**Affiliations:** Department of Internal Medicine, Section of Clinical Immunology and Allergy, University of Manitoba, Winnipeg, MB Canada

**Keywords:** Adult onset food allergy, Mast cell activation syndrome, Anaphylaxis

## Abstract

**Background:**

Adult onset food allergy is a rare, but increasingly recognized phenomenon. Mast cell activation syndromes present an ongoing diagnostic and classification challenge. The combination of the two has been rarely described in the literature.

**Case presentation:**

We present a case of a new onset, IgE mediated food allergy in combination with a mast cell activation syndrome in an elderly patient not known to have a history of atopy. He presented to a hospital with a first presentation of anaphylaxis manifesting profound hypotension following consumption of a stew consisting of fish and shellfish. He had a persistently elevated serum tryptase and demonstrated evidence of high titre serum specific IgE to shellfish. He responded well to histaminergic blockade.

**Conclusions:**

Given that mast cell activation syndromes pose an increased risk for recurrent, severe anaphylaxis and that secondary causes of mast cell activation syndromes are more prevalent with aging, this case highlights the importance of considering this entity when evaluating an elderly patient with a first presentation of anaphylaxis.

## Background

Food allergy is reported to affect approximately 2 % of the adult population, with the majority having had onset during childhood [1, 2]. There is an increasing recognition of adult onset food allergy, however these typically are associated with pollen-plant association, and are more often food-dependent, exercise-induced, anaphylaxis (FDEIAn) [3, 4]. Profound cardiovascular derangements during anaphylaxis suggest the possibility of an underlying mast cell activation syndrome and should prompt further investigation [5, 6]. The association between IgE mediated Hymenoptera anaphylaxis with hypotension and limited cutaneous findings and underlying mast cell activation disorders has been well established, and guideline recommendations include assessment of a serum tryptase, including in patients with negative skin prick testing, positive serum specific IgE who have had severe anaphylaxis [5].

According to recent consensus statements, mast cell activation syndromes are divided into three subtypes: Primary mast cell activation syndromes (MCAS), Secondary MCAS, and idiopathic MCAS. A mast cell activation disorder requires clinical symptomatology that is in keeping with the disorder, a transient, measurable increase in either serum tryptase or other markers of mast cell mediators and a response to agents that interfere with mast cell mediators [7, 8]. Recent attempts have been made to standardize an approach to suspected mast cell disorders, in order to appropriately classify individuals with evidence of mast cell activation that did not meet diagnostic criteria for systemic mastocytosis [7, 8].

The combination of adult onset food allergy with an underlying mast cell disorder has not been described previously in the literature, and this case report demonstrates the investigation of an elderly gentleman who presented with first onset anaphylaxis due to food ingestion with evidence of a suspected underlying mast cell activation syndrome.

## Case presentation

A 75-year-old male presented to the local emergency department exhibiting symptoms consistent with anaphylaxis. When found by his son in law he was flushed and unresponsive. On arrival of emergency medical personnel he was found to be hypotensive with blood pressure values of 80/60 mm Hg, hypothermic at 34.7 °C orally, and hypoxemic with a sPO_2_ of 88 % by pulse oximetry. He received a 500 cc IV normal saline bolus with EMS. In the emergency department he remained hypotensive with a blood pressure of 71/44 mm Hg. He was treated with 3000 cc of IV crystalloid, epinephrine 1:1000 0.3 mg IM once, diphenhydramine 25 mg IV with a second 50 mg IV dose, ranitidine 150 mg IV once, and methylprednisolone 250 mg IV once with improvement. He suffered a type 2 MI, which was felt to be related to anaphylaxis-associated hypotension after assessment by Cardiology. A serum tryptase value was not obtained by the emergency room physician at the time of his presentation.

He had no previous history of anaphylaxis, atopy, lymphoproliferative disorder or other neoplasm. His daily medications included aspirin 81 mg daily, and this, in addition to his other regular medications was continued post reaction. He did not take additional doses of aspirin, over the counter or herbal products on the day of reaction. He had not been started on any new medications. There was no family history of atopy. The Allergy and Clinical Immunology Service was contacted by the emergency physician, at which time the food intake history was unclear, and given his profound hypotension at presentation, a concern of a MCAS was raised. This is consistent with suggestions to investigate for MCAS in patient’s presenting with anaphylaxis with profound cardiovascular derangement and lacking documented urticaria, even if likely attributed to an IgE mediated reaction [5]. He was therefore discharged on cetirizine 10 mg orally daily, prednisone 50 mg orally for 5 days, diphenhydramine 25–50 mg orally q6 h as needed, and an epinephrine auto-injector. He was subsequently assessed in the Adult Allergy and Clinical Immunology outpatient clinic.

Between the anaphylactic episode and his appointment at the Allergy Clinic [approximate time 1 month] he consumed Atlantic cod without reaction. A food history obtained at his first visit revealed his initial event had developed following ingestion of a mixed fish and shellfish stew, which he had consumed without reaction on a regular basis. Following the meal he was alone in his room, until being found unresponsive by his son-in-law 3 h later. The patient could not recall the timeline of onset of symptoms, as his recall of the entire event was limited. Due to the concern of a potential underlying MCAS and the severity of his initial reaction it was felt safest to continue H1 receptor antagonist therapy, and due to the interference of anti-histamines on skin prick testing this was deferred for use of ImmunoCAP® [serum specific IgE (Phadia, Sweden)] for shellfish, finned fish and Hymenoptera venom. He was given an epinephrine auto-injector to be used in the event of subsequent anaphylactic reaction. At follow up his serum specific IgE was high positive for Shrimp at 13.7 kUA/L, and Crab at 7.3 kUA/L, moderately positive for Lobster at 2.9 kUA/L and Clam at 0.9 kUA/L, while testing negative with values <0.35 kUA/L for Salmon, Walleye Pike and Whitefish [9].

While both skin prick testing and serum specific IgE demonstrate the existence of clinical sensitization, they do not provide evidence for clinical allergy. Serum specific IgE has been reported to be in agreement with skin prick testing from between 50 and 90 % of the time, with average values between 70 and 75 %, and similar values in agreement between supervised challenges [10]. The interpretation of skin prick tests and serum specific IgE requires evaluation of historical features, physical examination and, at times, supervised challenge [10]. Although a supervised oral food challenge would have provided definitive evidence of an IgE mediated food allergy rather than sensitization, this was not undertaken due to the severity of his presentation with development of a myocardial infarction, his baseline limited myocardial reserve, and multiple comorbidities. It is acknowledged this limits the definitive confirmation of IgE mediated food allergy, but was made on a risk–benefit decision in the best interests of the patient.

A serum tryptase level drawn during his first appointment at the Allergy Clinic was 15 ng/ml [with a normal range from 1 to 11.4 ng/ml], an elevation not diagnostic of systemic mastocytosis, but suggestive of a mast cell activation disorder [11]. A creatinine obtained demonstrated a value of 92, corresponding to an eGFR of >60 ml/min, ruling out reduced renal clearance as a cause of accumulation of serum tryptase [12].

To further evaluate for a potential mast cell disorder, a 24-h urine methylhistamine level was obtained, demonstrating a value of 103 μg/g Cr (normal range 30–200 μg/g Cr). A serum protein electrophoresis demonstrated no evidence of an M protein. He was seen by the Adult Hematology/Oncology Service at Cancer Care Manitoba to definitively exclude a diagnosis of systemic mastocytosis, for which a bone marrow biopsy and c-KIT testing were completed. His c-KIT mutation testing was negative. A bone marrow biopsy demonstrated normal trilineage hematopoiesis with normal differentiation and maturation without definitive morphological evidence of mastocytosis or lymphoma, specifically revealing no large lymphoid aggregates, abnormal plasma cells, or spindle cells suggestive of mastocytosis. Accompanying flow cytometry demonstrated revealed a sample composed of 23 % lymphocytes, of which 84 % were T cells, 8 % NK cells, and there was a CD4/8 ratio of 0.9. Remaining cells were polyclonal B cells without evidence of lymphoma, plasma cell neoplasm, or mastocytosis. Tryptase was consistently elevated at 17 ng/ml on repeat testing.

Given his elevated tryptase, he was maintained indefinitely on cetirizine, and continued to avoid both fish and shellfish, but did require emergency department monitoring following administration of his epinephrine auto injector in January 2015 following ingestion of a perogy, of which the precise constituents were unknown, and development of a diffuse urticarial rash. He was treated with a 3 day course of 50 mg of oral prednisone.

He fulfills the proposed diagnostic criteria for diagnosis of a suspected MCAS based on guidelines published by Valent and colleagues, however we acknowledge the challenge of establishing the diagnosis in the context of a documented IgE mediated food allergy, and he may be best classified as a Secondary MCAS [IgE-dependent disease related] [7, 8, 13].

## Conclusions

The combination of food allergy with a mast cell activation syndrome with onset in the elderly population represents a novel combination that is not well described. It is unclear whether the patient in this case had an underlying mast cell disorder that was previously quiescent and was detected only due to the new development of an IgE mediated food allergy, or if sub-clinical sensitization to shellfish was able to manifest clinically with acquisition of a mast cell disorder. Theoretically the patient could have developed proliferation of a non-clonal mast cell population due to a subclinical entity such as malignancy, infection, subclinical thrombosis, an underlying autoimmune condition, increased stimulatory cytokines, or increased vasoactive peptides; as all are postulated triggers of mast cell activation [14].

The increasing prevalence of food allergy worldwide, and the heightened propensity for life threatening anaphylactic reactions in those with underlying mast cell reactivity highlights the importance of an educated, stepwise, evidence-based diagnostic approach to older adults presenting with anaphylaxis. The combination of IgE mediated allergy presenting with anaphylaxis associated with hypotension and underlying mast cell activation disorders is well established in Hymenoptera IgE hypersensitivity, and guideline recommendations to assess for serum tryptase exist in this situation [6]. According to a recent case series the absence of urticaria or angioedema with severe anaphylaxis and associated hypotension in response to Hymenoptera stings provides the strongest indicator of an underlying mast cell disorder [15]. This provides precedence for the combination of IgE mediated anaphylaxis and an underlying mast cell activation disorder, and the authors believe this case report highlights the importance of a high level of suspicion for mast cell disorders in patient’s presenting with anaphylaxis with profound cardiovascular manifestations and limited cutaneous manifestations, as has been previously described [5, 16].

A case such as this emphasizes the importance of an approach with thorough investigation of all potential allergen exposures, including those to which a patient has been exposed on multiple occasions without systemic or local reaction. This case suggests the need for further elucidation of patient characteristics that predispose to an underlying mast cell activation syndrome despite identification of an IgE mediated trigger, to better facilitate identification of patients who would benefit from additional intervention with mast cell stabilizing therapy.

It could be theorized that new sensitization to previously tolerated food antigens with resultant anaphylaxis could be associated with underlying mast cell activation disorders in adults without a history of atopy, particularly when presenting with profound hypotension. Further research is needed into the incidence of adult onset IgE mediated food allergies, anaphylaxis, and the rate at which it is associated with underlying mast cell activation, particularly given the association with MCAS and a heightened risk for a more severe anaphylactic reaction [5].

## Abbreviations

FDEIAn: food- dependent, exercise-induced, anaphylaxis
MCAS: mast cell activation syndrome
